# RP-HPLC Estimation of Risperidone in Tablet Dosage Forms

**DOI:** 10.4103/0250-474X.44601

**Published:** 2008

**Authors:** S. L. Baldania, K. K. Bhatt, R. S. Mehta, D. A. Shah

**Affiliations:** Anand Pharmacy College, Opp. Town Hall, Shri Ramkrishna Seva Mandal Campus, Anand-388 001, India; 1 A. R. College of Pharmacy, Vallabh Vidyanagar-388 120, India

**Keywords:** Reverse phase liquid chromatography, risperidone, RP-HPLC, specificity, validation

## Abstract

A simple, specific, accurate, and precise reverse phase liquid chromatographic method was developed and validated for the estimation of risperidone in tablet dosage forms. A Phenomenex Gemini C-18, 5 μm column having 250×4.6 mm i.d. in isocratic mode, with mobile phase containing methanol: acetonitrile: 50 mM potassium dihydrogen orthophosphate (80:10:10 v/v) was used. The flow rate was 1.3 ml/min and effluents were monitored at 234 nm. Clozapine was used as an internal standard. The retention time of risperidone and clozapine were 2.5 min and 3.3 min, respectively. The method was validated for linearity, accuracy, precision, specificity, limit of quantification, limit of detection, robustness and stability. The limit of detection and limit of quantification for estimation of risperidone was found to be 500 ng/ml and 990 ng/ml, respectively. Recovery of risperidone was found to be in the range of 99.02-101.68%. Proposed method was successfully applied for the quantitative determination of risperidone in tablet formulations.

Risperidone (RIS) is psychotropic agent used to treat schizophrenia, action of which is mediated through a combination of dopamine Type 2 (D_2_) and serotonin Type 2 (5HT_2_) receptor antagonism. It is a selective monoaminergic antagonist with high affinity for 5HT_2_, D_2_ and H1 histaminergic receptors^1^. It belongs to chemical class of benzisoxazole derivatives and is 3-[2-[4-[6-fluoro-1, 2-benzisoxazol-3-yl)-1-piperidinyl] ethyl]-6,7,8,9-tetrahydro-2-methyl-4H-pyrido-[1,2-a]-pyrimidin-4-one with molecular formula of C_23_ H_27_ FN_4_ O_2_ and molecular weight of 410.49.

Literature survey revealed that various methods have been reported for estimation of RIS in biological matrices such as plasma with help of LC^2^,^3^, LC with diode array detection^4^, LC with tandem mass spectrometry^5^–^7^ and LC with electrochemical detection^8^. Few stability-indicating methods have been reported for determination of RIS in bulk powder and tablets in presence of its degradation products^9^,^10^. However no method was reported for quantitation of RIS in tablet dosage forms in the literature. Present study involved development of a simple liquid chromatographic method for the estimation and quantitation of RIS in tablet dosage forms.

The Liquid chromatographic system consisted of following components: Shimadzu HPLC model (VP series) containing LC-10AT (VP series) pump, variable wavelength programmable UV/VIS detector SPD-10AVP and Rheodyne injector (7725i) with 20 μl fixed loop. Chromatographic analysis was performed using Spinchrom software on a Phenomenex Gemini C18 column with 250×4.6mm i.d. and 5 μm particle size. The Shimadzu electronic balance (AX 200) was used for weighing purpose.

An analytically pure sample of RIS and clozapine (CLO), were procured as gift sample from Torrent Pharmaceuticals Ltd (Ahmedabad, India). Analytical grade methanol (purity 99.9%) was procured from E. Merck (Ahmedabad). LC grade water was obtained by double distillation and purification through Milli-Q water purification system. Potassium dihydrogen orthophosphate (AR grade, purity 99.5%) was procured from Qualigens. Tablet formulation A, (Risdone, Intas Pharmaceutical Ltd., Ahmedabad) and B (Respidone, Torrent Pharmaceutical Ltd, Ahmedabad) was procured from a local pharmacy with labeled amount 2 mg RIS per tablet.

Potassium dihydrogen orthophosphate was weighed (0.68 g) and dissolved in 100 ml of water. This solution was mixed with 800 ml of methanol and finally 100 ml of acetonitrile was added to above prepared solution and mixed well. The solution was sonicated for 10 min and filtered using Whatman filter paper No.1.

A stock solution of RIS was prepared by accurately weighing 25 mg of drug, transferring to 25 ml volumetric flask, dissolving in 5 ml of methanol and diluting it up to the mark with methanol. Appropriate aliquot of this solution was further diluted to 10 ml with methanol to obtain final standard solution of 50 μg/ml of RIS. Resultant solution was filtered through Whatman filter paper No.1.

A stock solution of CLO was prepared by accurately weighing 25 mg of the drug, transferring to 25 ml volumetric flask, dissolving in 5 ml of methanol and diluting it up to the mark with methanol. Appropriate aliquot of this solution was further diluted to 10 ml with methanol to obtain final standard solution of 100 μg/ml of CLO. Resultant solution was filtered through Whatman filter paper No.1.

Twenty tablets were accurately weighed and finely powdered. Tablet powder equivalent to 10 mg of RIS was taken in 50 ml of volumetric flask containing methanol (approximately 20 ml) and was shaken occasionally to dissolve the drug and filtered through Whatman filter paper No. 1. The filter paper was washed with more solvent collecting the filtrate. The filtrate volume was adjusted to the mark with the same solvent to obtain concentration of 200 μg/ml. The resulting solution was filtered through Whatman filter paper No.1. Appropriate aliquotds of this solution was taken in 10 ml of volumetric flask; 1 ml of standard CLO stock solution added and finally diluted to 10 ml with mobile phase to obtain final concentration of 1 μg/ml of RIS and 10 μg/ml of CLO, respectively. The resulting solution was again filtered using Whatman filter paper No.1 and then was sonicated for 10 min.

A reverse phase C-18 column equilibrated with mobile phase methanol:acetonitrile:50 mM potassium dihydrogen orthophosphate (80:10:10,v/v) was used. Mobile phase flow rate was maintained at 1.3 ml/min and effluents were monitored at 234 nm. The sample was injected using a 20 μl fixed loop, and the total run time was 10 min.

Appropriate aliquots of standard RIS stock solution (0.5 μg/ml) were taken in different 10 ml volumetric flasks, followed by addition of 1ml of standard CLO solution (100 μg/ml) and resultant solution was diluted up to the mark with mobile phase to obtain final concentration of 1, 3, 5, 7, 9 and 11 μg/ ml of RIS and 10 μg/ ml of CLO, respectively. These solutions were injected into chromatographic system and chromatograms were obtained and peak area ratio was determined for each concentration of drug solution. Calibration curve of RIS was constructed by plotting peak area ratio vs. applied concentration of RIS and regression equation was computed. Similarly the sample solution was chromatographed and concentration of RIS in tablet samples was found out using regression equation.

The method was validated for linerarity, range, accuracy, precision, specificity, detection limit, quantitation limit and robustness by following procedures. The accuracy of the method was determined by calculating recovery of RIS by method of standard addition. Known amount of RIS (0, 1, 4, 10 μg/ml) was added to a pre quantified sample solution, and the amount of RIS was estimated by measuring the peak area ratios and by fitting these values to the straight-line equation of calibration curve.

The intraday and inter day precision study of RIS was carried out by estimating the corresponding responses 3 times on the same day and on 3 different days for the period of 1 week for 3 different concentrations of RIS (1, 5, 11 μg/ml). Repeatability studies were carried out by estimating response of 3 different concentrations of RIS (1, 5, 11 μg/ml) in triplicate. The linearity of the method was determined at six concentration levels ranging from 1-11 μg/ml for RIS.

Commonly used excipients that include, dibasic calcium phosphate dihydrate, microcrystalline cellulose, magnesium stearate and polyethylene glycol were spiked into a preweighed quantity of drug. The chromatogram was taken by appropriate dilutions and the quantity of drug was determined. A calibration curve was prepared using concentrations in the range of 0.5-1.0 μg/ml (expected detection limit range). The standard deviation of y-intercepts of regression line was determined and kept in following equation for the determination of detection limit and quantitation limit. Detection limit = 3.3σ/s; Quantitation limit = 10σ/s, where σ is the standard deviation of y-intercepts of regression lines and s is the slope of the calibration curve.

In order to demonstrate the stability of both standard and sample solutions of RIS during analysis, both the solutions were analyzed over a period of 24 h at room temperature and then analyzed. Robustness of the method was studied by changing the composition of organic phase by ±5% and pH by 0.2, and also by observing the stability of the drug for 24 hr at ambient temperature in mobile phase.

UV overlain spectra of both RIS and CLO showed that both the drugs absorbs appreciably at 234 nm, so 234 nm was selected as the detection wavelength in liquid chromatography (fig. 1). Optimization of mobile phase was performed based on resolution, asymmetric factor and peak area obtained. Different mobile phases were tried but satisfactory separation, well resolved and good symmetrical peaks were obtained with the mobile phase methanol: acetonitrile: 50 mM KH_2_PO_4_ (80:10:10, v/v). The retention time of RIS was found to be 2.5 min and that of CLO was found to be 3.3 min, respectively (fig. 2). Resolution between RIS and CLO was found to be 4.0, which indicate good separation of both the compounds. The asymmetric factor for RIS was 1.6. The calibration curve for RIS was obtained by plotting the peak area ratio versus the concentration of RIS over the range of 1-11 μg/ml, and it was found to be linear with r^2^ = 0.999. The data of regression analysis of the calibration curves are shown in Table 1. Detection limit for RIS was 0.5 μg/ml and quantitation limit was 0.9 μg/ml, which suggest that a nanogram quantity of both the compounds can be estimated accurately. The validation parameters are summarized in Table 2. The results for stability studies revealed that for the solutions, retention time and peak area of RIS and internal standard remained almost unchanged and no significant degradation was observed within the indicated period. The recovery of RIS was found to be in the range of 99.02-101.68%. The system suitability test parameters are shown in Table 3. The proposed liquid chromatographic method was applied for the determination of RIS in tablet formulations (A and B). The result for RIS was comparable with the corresponding labeled amount (Table 4).

**Fig. 1:**
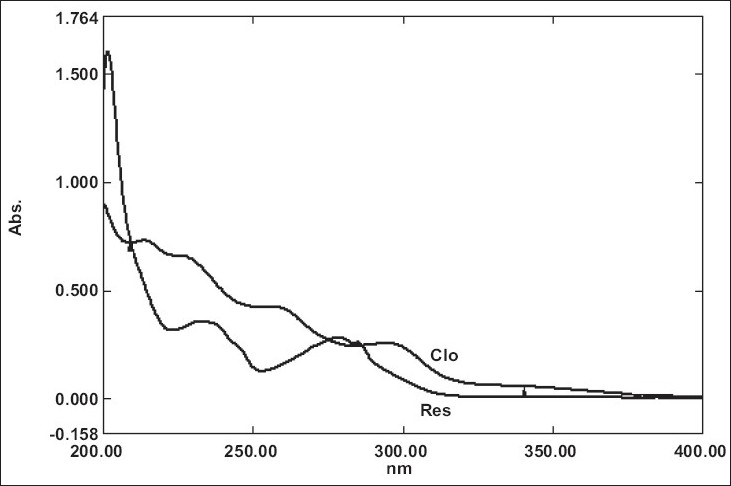
Overlaid UV spectra of RIS (10 μg/ml) and CLO (10 μg/ml) ^b^mean value±standard deviation of three determinations; Tablet A: Risdone (Intas {Pharmaceutical Ltd. India) Tablet B: Respidone (Torrent Pharmaceutical Ltd. India) each containing labeled amount of 2 mg of RIS; RIS is risperidone.

**Fig. 2:**
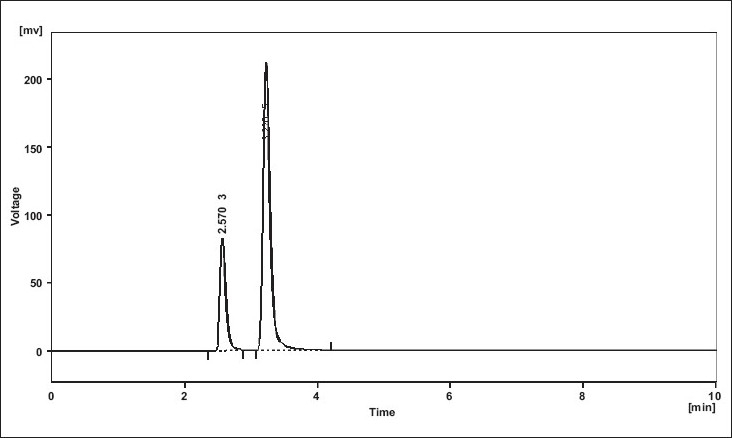
Typical HPLC chromatogram of RIS (RT 2.5 min) and CLO (RT 3.3 min) HPLC chromatogram showing well resolved peaks of risperidone and clozapine (used as internal standard) on C18 Phenomenex Gemini column using methanol: acetonitrile: 50 mM potassium dihydrogen orthophosphate (80:10:10, v/v) as mobile phase.

**TABLE 1:** REGRESSION ANALYSIS OF THE CALIBRATION CURVES FOR THE PROPOSED METHOD

Parameters	Values
Calibration range (μg/ml)	1 - 11 μg/ml
Slope	0.036
Standard deviation of slope	0.001137
Intercept	0.00908
Standard deviation of intercept	0.00572
Correlation coefficient (r)	0.9990

**TABLE 2:** SUMMARY OF VALIDATION PARAMETERS FOR THE PROPOSED METHOD

Parameters	Values
Detection limit (μg/ml)	0.500
Quantitation limit (μg/ml)	0.900
Accuracy (%)	99.02-101.68
Precision (RSD^a^,%)	
Intraday (n=3)	0.20-0.82
Interday (n=3)	0.18-1.50
Repeatability (RSD^a^, n=3)	0.14-0.72

a RSD indicates relative standard deviation

**TABLE 3:** SYSTEM SUITABILITY TEST PARAMETERS FOR RIS BY THE PROPOSED METHOD

System suitability parameters	Values
Retention time (min)	2.5
Resolution	4.0
Tailing factor (asymmetric factor)	1.60

**TABLE 4:** ASSAY RESULTS OF TABLET FORMULATIONS USING PROPOSED METHOD

Formulations	Labelled Amount (mg)	Amount obtained (mg)^b^	% Recovery^b^
A	2	1.98±0.026	99.49±0.76
B	2	2.02±0.015	101.1±1.15

b Mean value±standard deviation of five determinations; Tablet A: Risdone (Intas Pharmaceutical Ltd.India) Tablet B: Respidone (Torrent Pharmaceutica Ltd. India).

Proposed study describes new LC method for the estimation of RIS in tablet formulations. The method was validated and found to be simple, sensitive, accurate and precise. Percentage of recovery shows that the method is free from interference of the excipients used in the formulation. Therefore the proposed method can be used for routine analysis for estimation of RIS in its tablet formulations.
